# Serological Tests Do Not Predict Residual Fibrosis in Hepatitis C Cirrhotics with a Sustained Virological Response to Interferon

**DOI:** 10.1371/journal.pone.0155967

**Published:** 2016-06-15

**Authors:** Roberta D’Ambrosio, Elisabetta Degasperi, Alessio Aghemo, Mirella Fraquelli, Pietro Lampertico, Maria Grazia Rumi, Floriana Facchetti, Eleonora Grassi, Giovanni Casazza, William Rosenberg, Pierre Bedossa, Massimo Colombo

**Affiliations:** 1 Division of Gastroenterology and Hepatology, Fondazione IRCCS Cà Granda Ospedale Maggiore Policlinico di Milano, Università degli Studi di Milano, Milan, Italy; 2 Division of Gastroenterology and Endoscopy, Fondazione IRCCS Cà Granda Ospedale Maggiore Policlinico di Milano, Milan, Italy; 3 Division of Hepatology, Ospedale San Giuseppe, Università degli Studi di Milano, Milan, Italy; 4 Department of Biomedical and Clinical Sciences, Luigi Sacco, Università di Milano, Milan, Italy; 5 Centre for Hepatology, Division of Medicine, University College of London, London, United Kingdom; 6 Department of Pathology and INSERM U773, Beaujon Hopital, Universitée Paris-Diderot, Clichy, France; Università degli Studi di Palermo, ITALY

## Abstract

**Background and Aim:**

Liver biopsy (LB) has lost popularity to stage liver fibrosis in the era of highly effective anti-hepatitis C virus (HCV) therapy, yet diagnosis of persistent cirrhosis may have important implications following HCV eradication. As performance of serological non-invasive tests (NITs) to predict residual fibrosis in *non-viremic* HCV patients is unknown, we investigated accuracy of NITs to predict residual fibrosis in cirrhotics after a sustained virological response (SVR) to interferon (IFN).

**Methods:**

Thirty-eight patients with a pre-treatment histological diagnosis of cirrhosis and a 48–104 months post-SVR LB were tested with APRI, CDS, FIB-4, FibroQ, Forns Score, GUCI Index, King Score, Lok Index, PLF, ELF. In 23 (61%) patients, cirrhosis had histologically regressed.

**Results:**

All NITs values declined after SVR without any significant difference between regressors and non-regressors (AUROC 0.52–0.75). Using *viremic* cut-offs, PPV ranged from 34% to 100%, with lower NPV (63% - 68%). NITs performance did not improve using derived cut-offs (PPV: 40% - 80%; NPV: 66% - 100%). PLF, which combines several NITs with transient elastography, had the best diagnostic performance (AUROC 0.75, Sn 61%, Sp 90%, PPV 80%, NPV 78%). After treatment, none of the NITs resulted significantly associated with any of the histological features (activity grade, fibrosis stage, area of fibrosis).

**Conclusions:**

The diagnostic estimates obtained using both *viremic* and derived cut-off values of NITs were suboptimal, indicating that none of these tests helps predicting residual fibrosis and that LB remains the gold standard for this purpose.

## Introduction

The long-standing dogma that cirrhosis is an irreversible condition has been repeatedly challenged in last decades. Indeed many studies in hepatitis C (HCV) patients with a sustained virological response (SVR) to Interferon (IFN)-based regimens showed cirrhosis regression in 30% to 60% patients 3 to 5 years after SVR **[1–16]**; this histological outcome was associated with reduced risk of developing hepatocellular carcinoma (HCC), hepatic decompensation and variceal bleeding, while, on the contrary, a clinical benefit was not observed in SVR patients with residual cirrhosis **[9]**. These findings rose the argument about need for assessing residual cirrhosis in HCV patients with an SVR, in order to deliver accurate prognosis and optimize clinical management. Indeed, all SVR cirrhotic patients are currently recommended to keep lifelong surveillance for HCC with abdominal ultrasound (US) every 6 months **[17]**, as these patients still carry a 0.6%-1.2% yearly risk of HCC **[13,16,18,19]**. The demonstration of cirrhosis regression would allow for tailored surveillance intervals, as the reduced risk of HCC in cirrhosis regressors would make US surveillance every 6 months non cost/effective.

Liver biopsy (LB) currently represents the gold standard for accurately assessing liver fibrosis stage and consequently identifying cirrhosis regression, however it has limited application because it is an invasive procedure, poorly accepted by patients and ultimately carries a risk of complications **[20,21]**. Moreover, LB accuracy is biased by heterogeneous deposition of fibrosis in the liver, sampling error and inter-observer variability **[22,23]**. These drawbacks highlight the need for alternative non-invasive methods to assess fibrosis stage following an SVR. In this setting, we previously demonstrated that transient elastography (TE) has low diagnostic accuracy in identifying residual cirrhotic patients after an SVR, mainly as a consequence of fibrosis remodeling **[24]**. The aim of this study was to investigate the diagnostic performance of most popular serological markers of hepatic fibrosis in a unique albeit small group of patients with histologically documented cirrhosis who underwent a second liver biopsy 5 years after achieving an SVR to IFN-based therapies.

## Material and Methods

### Patient population

This study is a *post-hoc* analysis of a cohort study of 38 HCV cirrhotic patients, who underwent a LB following an SVR to assess histological modifications associated with viral clearance **[12]**. The first LB was performed within 12 months before anti-HCV treatment, while the second at least 4 years after the SVR [**12]**. Exclusion criteria were HBV or HIV co-infection, >40 g/day alcohol intake, malignancies, contraindication to LB. At the time of post-SVR LB, all patients had liver stage concomitantly assessed through TE and the following serological non-invasive tests (NITs): APRI, CDS, FIB-4, FibroQ, Forns Score, GUCI Index, King Score, Lok Index, PLF and ELF (*see* Material and Methods Section). All patients gave their written informed consent to use of their medical records and perform the post-treatment LB for the study, which was approved by the Institutional Review Board of the Department of Internal Medicine.

### Histological assessment

All pre-treatment LBs were performed with a cutting needle (Tru-cut 16 G, TSK Laboratories, Tokyo, Japan), whilst a Menghini-like semiautomatic biopsy device (Biomol 16 G, HS Hospital Service, Latina, Italy) was used for post-treatment samples. Liver biopsies were considered adequate for histological analysis if longer than 10 mm and/or carrying at least 12 portal tracts. All biopsies were read independently by two expert Pathologists blinded to any clinical information. Fibrosis stage was assessed according to the METAVIR scoring system **[25]**, while hepatic inflammation was evaluated according to both METAVIR and Ishak grading systems **[25,26].** Cirrhosis regression was defined as decrease from F4 to F3 or less when comparing pre- and post-treatment LBs **[12]**. Area of fibrosis was calculated by using the HISTOLAB® software (ALPHELYS, Plaisir, France), and was defined as ratio of fibrous tissue area stained with picrosirius red (%) upon the total tissue surface, as previously described **[12]**.

### Non-invasive measurements

#### Transient elastography

Transient elastography was performed as already described **[27]**; liver stiffness measurement (LSM) was expressed in kiloPascal (kPa) as the median value of the successful measurements. As previously published **[28]** TE threshold of ≥ 12 kPa was used for the diagnosis of cirrhosis.

#### Serological non-invasive tests

The following serological indirect and direct markers of liver fibrosis **[29]** were tested according to their formula:

1. Indirect serological markers
  APRI (AST to platelet ratio): AST levels (× ULN)/platelets count (10^3^/l) × 100
  CDS (Cirrhosis Discriminate Score): calculated by summing the scores awarded for the following laboratory results (Table 1)

**Table 1:** Cirrhosis Discriminate Score (CDS) calculation.

Parameters	0	1	2	3	4	5	6
INR	<1.1	1.1–1.4	>1.4				
ALT/AST	>1.7	1.7–1.2	1.19–0.6	<0.6			
PLT /mm^3^	>340	340–280	279–220	219–160	159–100	99–40	<40
  FIB-4: [age (yr) × AST (U/l)]/[(PLT (10^9^/l)] × [ALT (U/l)1/2]
  FibroQ: [(10 x age (yr)) x AST (U/l) x PT INR)/[PLT (10^9^/l) x ALT (U/l)]
  Forns Score: 7.811–3.131 × ln [PLT (10^9^/l)] × 0.781 ln [GGT (U/l)] + 3.467 × ln [age (yr)] - 0.014 [cholesterol (mg/dl)]
  GUCI (Goteborg University Cirrhosis Index): (AST/ULN) × INR × 100/PLT (10^9^/l)
  King Score: age (yr) × AST (U/l) × INR/PLT (10^9^/l)
  Lok Index:– 5.56–0.0089 × PLT (10^3^/mm) + 1.26 × AST/ALT ratio + 5.27 × INR
  PLF (Predicted Liver Fibrosis): 0.956 + 0.084 × TE –0.004 × King Score + 0.124 × Forns Score + 0.202 × APRI Score.
  Reference cut-offs for cirrhosis, obtained from literature, were APRI >1.5, CDS ≥8, Fib4 >3.25, FibroQ >2.6, Forns Score ≥6.9, GUCI >0.26, King Score ≥16.7, Lok Index >0.5 and PLF ≥2.98 **[30–39]**.
2. *Direct serological markers*. Enhanced Liver Fibrosis (ELF) test combines semiquantitative serum measurements of tissue inhibitor of metallo-proteinases-1 (TIMP-1), amino-terminal propeptide of type III collagen (PIIINP) and hyaluronic acid (HA). ADVIA Centaur CP immunochemical analyzer was used according to manufacturer’s instructions and the ELF score was calculated by the following equation: -7.412 + [ln(HA)*0.681] + [ln(PIIINP)*0.775] + [ln(TIMP-1)*0.494] + 10 **[40–43]**. Four cut-offs (9.3, 9.8, 10.3, 11.3) were used for the diagnosis of cirrhosis (F4), since a univocal standard threshold for F4 has not been validated **[41–44]**.

### Statistical analysis

Comparisons between groups were made by using the Mann–Whitney U or the Student’s-t tests for continuous variables, and the χ2 or Fisher test for categorical data. A probability value of p<0.05 was considered statistically significant. The diagnostic accuracy of non-invasive markers in SVR patients was expressed as sensitivity, specificity, positive and negative likelihood ratios (LR+ and LR-), positive and negative predictive values (PPV and NPV), using receiver operating characteristic (ROC) curves analysis. The accuracy of NIT and TE to identify cirrhosis has been assessed. A receiver operating characteristic (ROC) curve analysis was performed and the area under the ROC curve (AUC) has been reported.

Categorical data were reported as counts (percentages) and continuous variables as mean (standard deviation) or median (interquartile range, IQR), as appropriate.

Uni- and multivariate linear regression analyses were performed to assess the ability of the NITs (APRI, CDS, FIB-4, FIBRO-Q, FORNS score, GUCI index, King score, Lok index, ELF, PLF) in predicting the histological features (i.e. activity grade, fibrosis stage, area of fibrosis). Also TE was included in the analysis. Only those parameters that showed a statistically significant result at the univariate analysis were entered in the multivariate analysis. The performance of the linear models was evaluated by the determination coefficient (R^2^). Two analyses were performed considering, in separate, pre- and post-treatment (SVR) data. P values lower than 0.05, two sided, were considered statistically significant. All statistical analyses were performed with SAS statistical software (release 9.4; SAS Institute Inc, Cary, NC).

## Results

### Patient population

Clinical data were available at baseline and at the time of second LB, 61 (48–104) months after the SVR. Thirty-three (89%) patients had also a post-SVR LSM, while none had a baseline TE assessment. At post-SVR LB, 35 (92%) patients showed normal transaminases whereas 36 (95%) had a platelet count higher than 100,000/mm^3^. Post-SVR liver biopsies showed cirrhosis regression in 23 (61%) patients: F3 in 14 (61%), F2 in 7 (30%), and F1 in 2 (9%). Cirrhosis persisted in the remaining 15 (39%). No significant clinical differences were observed between patients staged F4 or <F4, after treatment (**Table 2)**.

**Table 2:** Patients' characteristics at the time of post-SVR liver biopsy.

Feature	Overall	Regressors	Non-Regressors	p-value^§^
	(n = 38)	(n = 23)	(n = 15)	
Males*	24 (63%)	15 (65%)	9 (60%)	1.0
Age, years**	66 (46–75)	64 (46–75)	64 (56–72)	0.98
Body weight, Kg**	75 (50–98)	73 (52–90)	73 (55–93)	0.53
BMI > 25 kg/m^2^ *	16 (42%)	12 (52%)	6 (40%)	0.60
pnALT^±^*	35 (92%)	21 (91%)	14 (93%)	1.0
PLT > 100x10^3^/mm^3^*	36 (95%)	22 (96%)	15 (100%)	1.0
INR < 1.2	38 (100%)	23 (100%)	15 (100%)	1.0
Liver core size, mm**	30 (10–45)	30 (15–50)	30 (10–45)	0.22
Fibrosis stage				0.31
F0	0 (0%)	0 (0%)	0 (0%)	
F1	2 (5%)	2 (9%)	0 (0%)	
F2	7 (18%)	7 (30%)	0 (0%)	
F3	14 (37%)	14 (61%)	0 (0%)	
F4	15 (40%)	0 (0%)	15 (100%)	

^**§**^ p-value: regressors *vs*. non-regressors

*number (%)

** median (range)

^±^ pn: persistent normal

### Indirect markers of fibrosis

NITs values at post-SVR LB and their reference cut-offs for the diagnosis of cirrhosis are shown in **Table 3**. Forns Score and PLF were calculated in 33 (87%) patients with available cholesterol and TE values.

**Table 3:** Median^#^ NIT values at post-SVR liver biopsy and reference cut-offs for the diagnosis of cirrhosis.

Test	Reference	Overall	Regressors	Non-Regressors	p-value
	Cut-off for F4	(n = 38)	(n = 23)	(n = 15)	
**APRI**	**≥ 1.5**	**0.3 (0.2–0.9)**	**0.3 (0.2–0.9)**	**0.3 (0.2–0.8)**	**0.48**
**CDS**	**> 8**	**5 (2–7)**	**5 (2–6)**	**5 (3–7)**	**0.49**
**FIB-4**	**> 3.25**	**1.7 (0.8–3.7)**	**1.7 (0.8–2.7)**	**1.7 (1.1–3.7)**	**0.38**
**FibroQ**	**> 2.6**	**3.9 (1.5–7.9)**	**3.9 (1.5–6.3)**	**4.0 (1.7–7.9)**	**0.26**
Forns Score*	**> 6.9**	**5.1 (3.1–8.5)**	**5.1 (3.1–8.5)**	**5.3 (4.0–8.0)**	**0.83**
**GUCI Index**	**> 0.26**	**0.4 (0.2–1.0)**	**0.4 (0.18–0.95)**	**0.4 (0.21–0.90)**	**0.56**
**King Score**	**> 16.7**	**7.7 (3.8–20.6)**	**7.7 (3.8–16.9)**	**7.7 (4.7–20.6)**	**0.48**
**Lok Index**	**> 0.5**	**0.4 (0.1–0.7)**	**0.4 (0.1–0.6)**	**0.4 (0.1–0.7)**	**0.24**
PLF*	**> 2.98**	**2.5 (1.7–5.0)**	**2.5 (1.7–5.0)**	**2.5 (2.2–4.7)**	**0.01**
ELF**	**>9.3, >9.8**	**8.6 (6.8–10.0)**	**8.6 (7.0–10.0)**	**8.4 (6.8–9.9)**	**0.70**
	**>10.3, >11.3**				

^**#**^ Results are reported as median (range) values

* Calculated in 33 patients for whom valid TE assessments and/or cholesterol values were available

** Calculated in 29 patients

**Fig 1:**
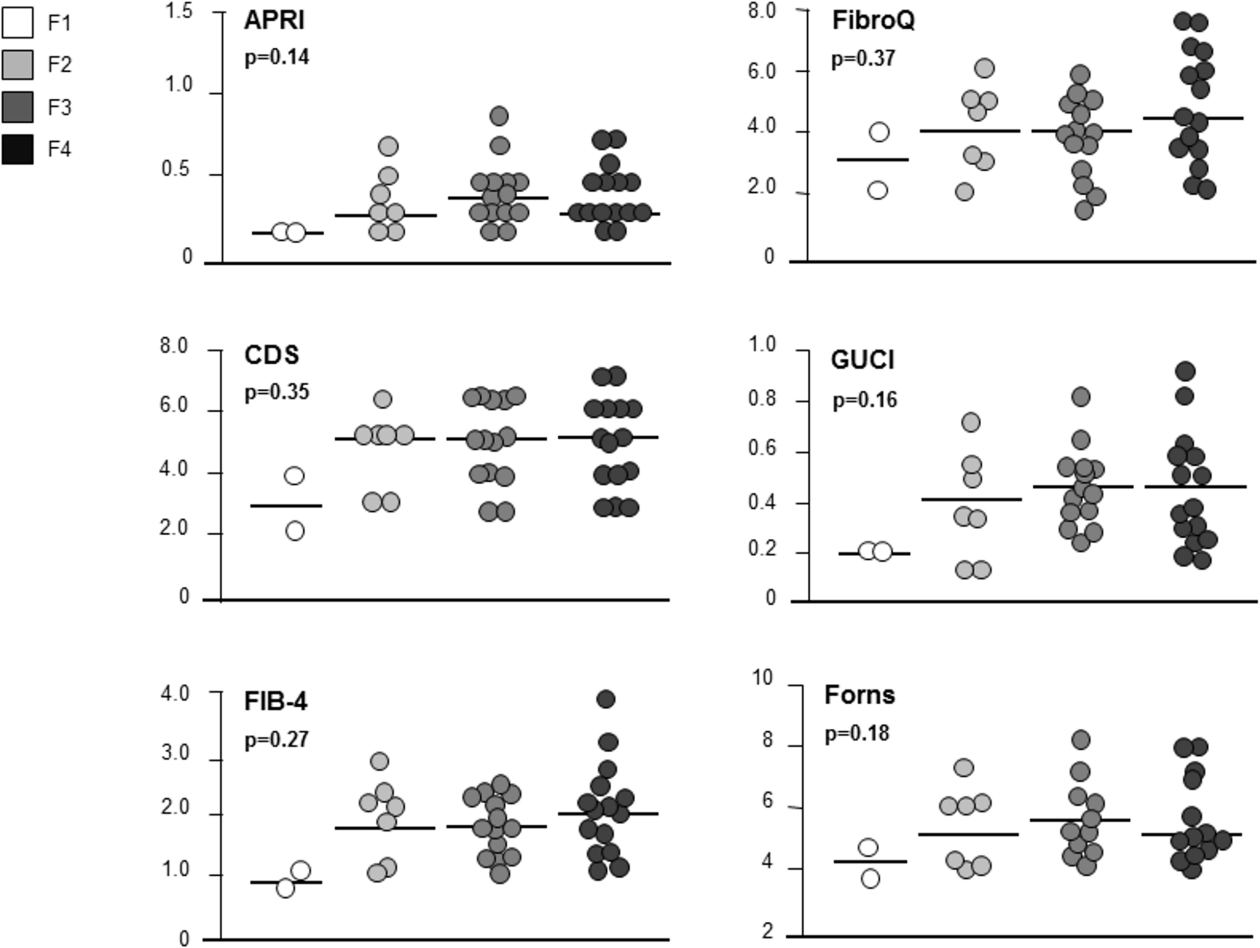
Non-invasive Tests (NITs) values stratification according to post-SVR fibrosis stage.

**Fig 2:**
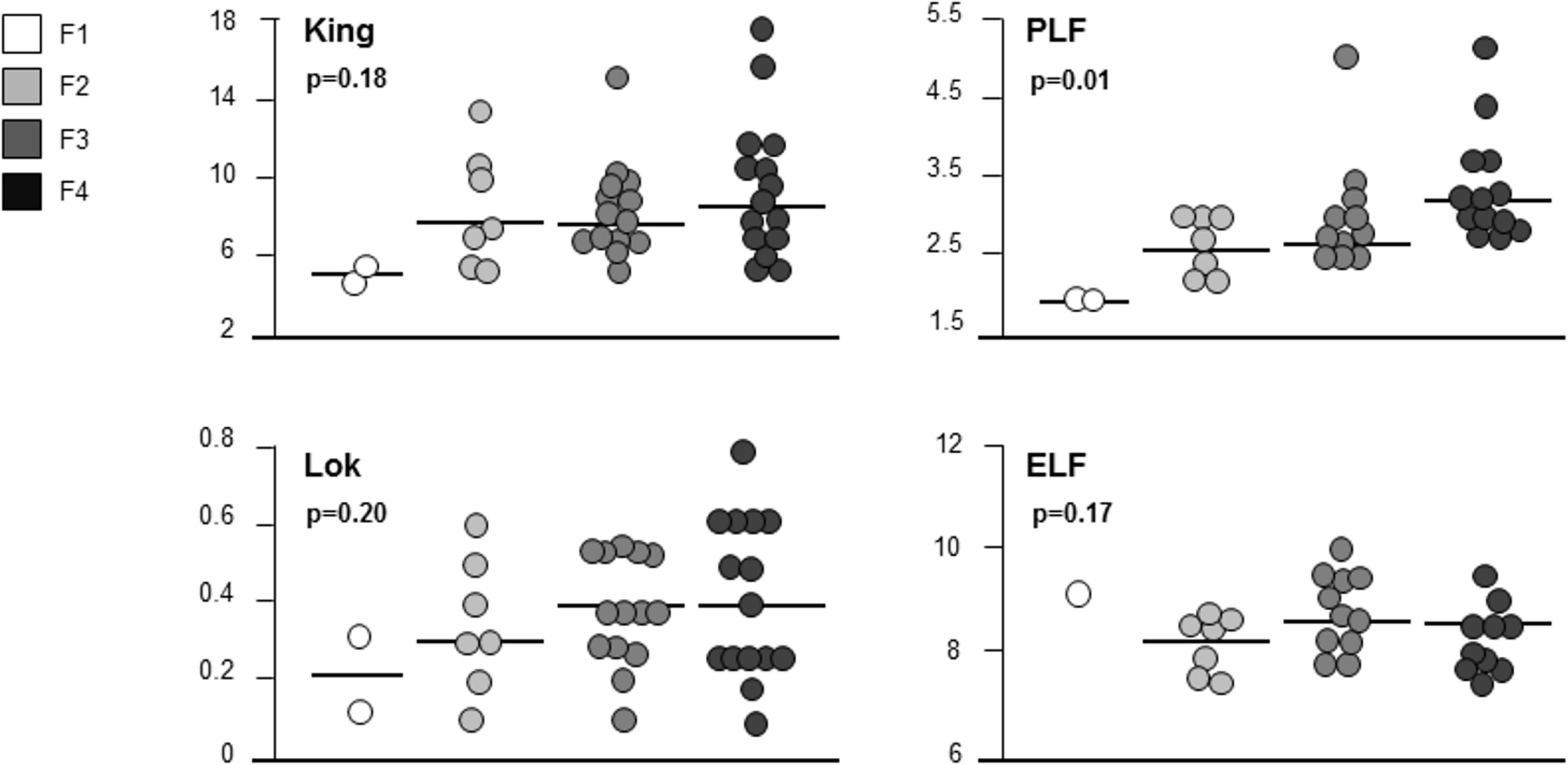
Non-invasive Tests (NITs) values stratification according to post-SVR fibrosis stage.

Apart from PLF, NITs values did not significantly differ in regressed and non-regressed patients **(Table 3)**, and also when stratifying data according to post-SVR fibrosis stage [(F1 *vs*. F2 *vs*. F3 *vs*. F4): APRI p = 0.14, CDS p = 0.35, FIB-4 p = 0.27, FibroQ p = 0.37, Forns Score p = 0.18, GUCI p = 0.16, King Score p = 0.18, Lok p = 0.20], due to an extensive overlap of NITs values across all stages of fibrosis **(Figs 1 and 2)**. PLF was the only NIT to show significant differences when comparing fibrosis stages at post-SVR LB (F4 *vs*. <F4: p = 0.01; F4 *vs*. F3 *vs*. F2 *vs*. F1: p = 0.01) **(Table 3, Fig 2)**.

When analyzing diagnostic performances according to ROC curves generated on reference cut-offs, all NITs demonstrated low diagnostic accuracy in identifying patients with residual cirrhosis after the SVR. Indeed, due to low sensitivity and specificity, AUROC ranged from 0.51 (CDS) to 0.75 (PLF) **(Table 4)**. NITs accuracy remained low also when using the derived thresholds, obtained by combining the best sensitivity/specificity, with PPV values ranging between 40% (CDS) and 80% (FIB-4 and PLF), and NPV between 66% (APRI and Lok Index) and 100% (CDS) **(Table 4)**. Therefore, 5% to 40% of SVR patients were misclassified as regressors (<F4) by NITs, despite cirrhosis persistence at post-SVR LB.

**Table 4:** Non-invasive tests (NITs) diagnostic accuracy for the identification of patients with residual cirrhosis.

Test	Cut-off	Patients	Sens	Spec	LR+	LR-	PPV	NPV	AUROC
		(n)	(%)	(%)			(%)	(%)	(95%CI)
APRI	**> 0.5**	**9**	**91**	**27**	**3.0**	**0.8**	**47**	**66**	0.58 (0.39–0.75)
	>1.5	**0**	-	-	-	-	-	**-**	
CDS	**> 2**	**37**	**100**	**4.3**	**1.03**	**0.001**	**40**	**100**	0.51 (0.35–0.68)
	>8	**0**	-	-	-	-	-	-	
FIB-4	**> 2.3**	**7**	**27**	**96**	**6.1**	**0.7**	**80**	**67**	0.59 (0.41–0.74)
	> 3.25	2	6	100	-	0.9	100	62	
FibroQ	**> 5.2**	**10**	**40**	**87**	**3.0**	**0.60**	**67**	**69**	0.58 (0.41–0.74)
	> 2.6	29	80	26	1.1	0.7	41	67	
Forns Score	**> 6.4**	**7**	**38**	**90**	**3.8**	**0.6**	**71**	**69**	0.56 (0.37–0.73)
	> 6.9	6	23	90	2.3	0.8	60	64	
Guci Index	**> 0.52**	**9**	**33**	**87**	**2.5**	**0.7**	**62**	**68**	0.56 (0.39–0.72)
	> 0.26	29	80	26	1.1	0.7	41	68	
King Score	**> 10.1**	**9**	**46**	**87**	**3.1**	**0.6**	**68**	**69**	0.59 (0.40–0.75)
	> 16.7	3	13	100	-	0.8	100	63	
Lok Index	**> 0.5**	**9**	**27**	**91**	**3.0**	**0.8**	**67**	**66**	0.57 (0.40–0.73)
PLF	**> 2.6**	**14**	**61**	**90**	**5.8**	**0.4**	**80**	**78**	0.75 (0.57–0.89)
	> 2.98	**4**	31	95	6.1	0.7	80	68	
ELF Score	**> 8.1**	**18**	**60**	**74**	**2.3**	**0.5**	**54**	**78**	0.63 (0.43–0.80)
	> 9.8	2	90	10	1.1	0.9	34	67	

Sn: Sensitivity; Sp: Specificity; LR+: positive likelihood ratio; LR-: negative; likelihood ratio; PPV: positive predictive value, NPV: negative predictive value.

In bold are reported the diagnostic estimates according to the cut-off maximizing sensitivity and specificity derived from present data. In plain text the results according to the cut off reported in the literature.

**Fig 3:**
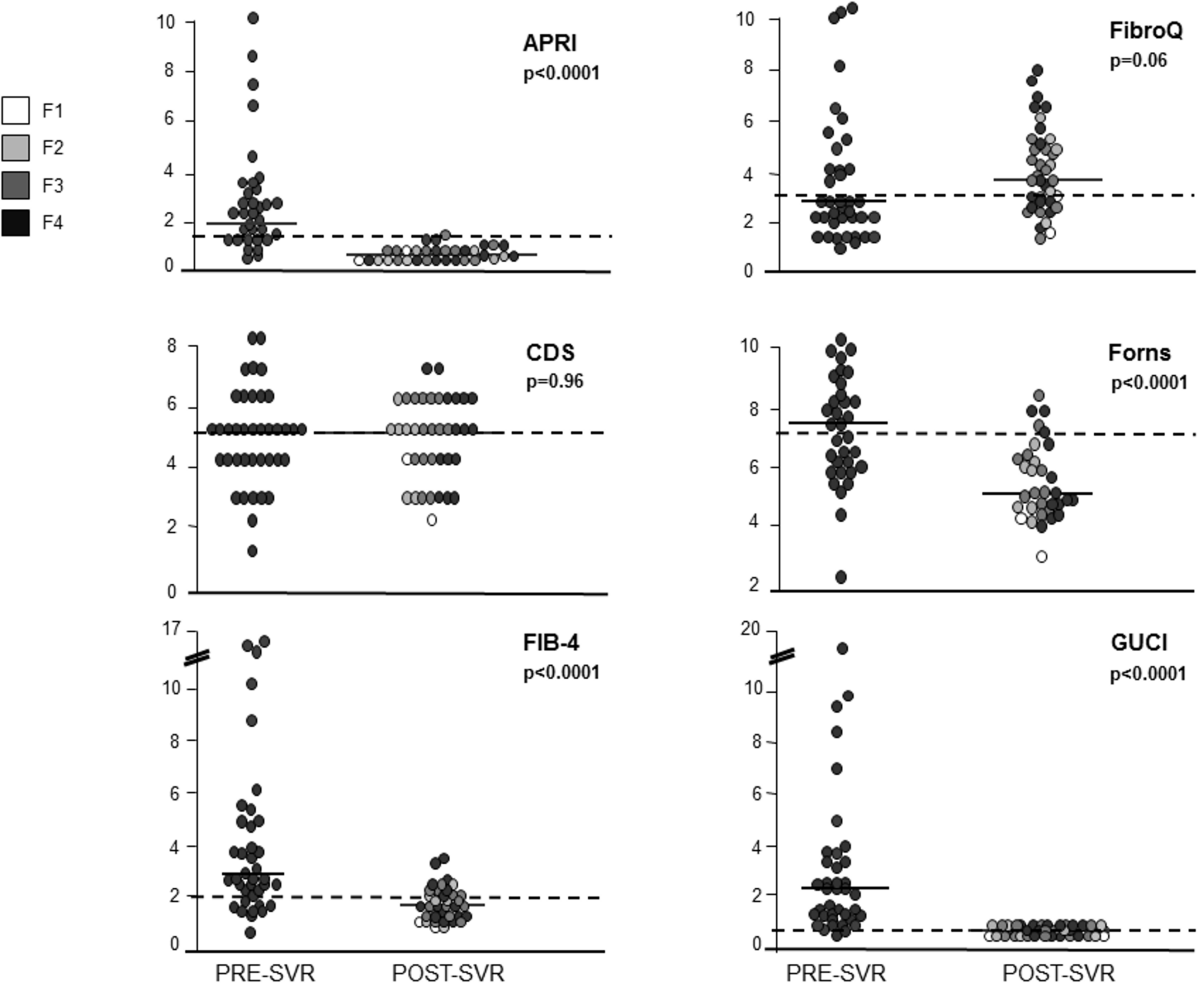
Non-invasive tests (NITs) values distribution at baseline and after an SVR.

**Fig 4:**
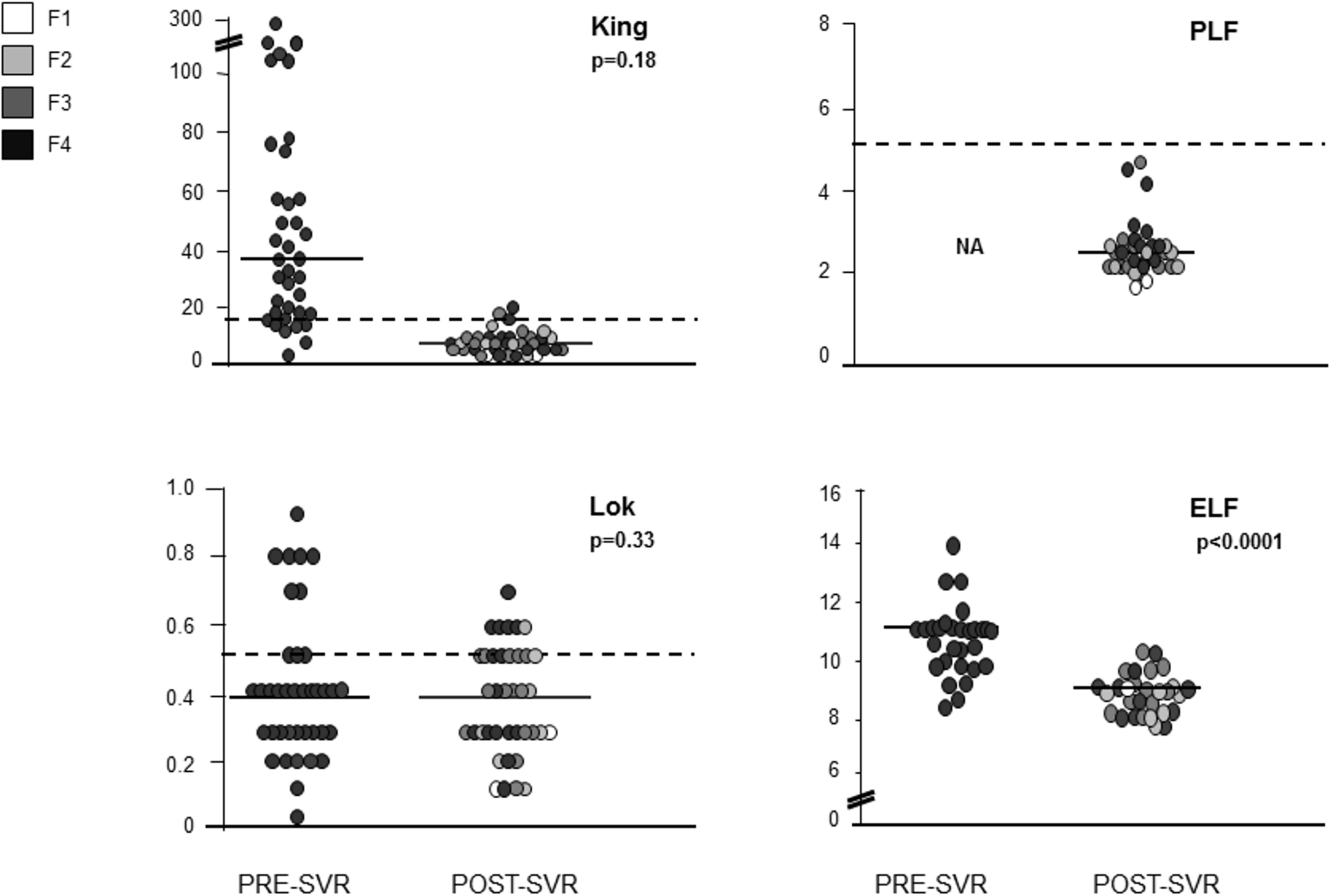
Non-invasive tests (NITs) values distribution at baseline and after an SVR.

NITs values at baseline are reported in Table 5. Most NITs showed a significant post-treatment decrease (Table 5; Figs 3 and 4), except for CDS, FibroQ and Lok Index.

**Table 5:** Non-invasive tests (NITs) values before and after treatment.

Test	Baseline	SVR	p-value
APRI	2.0 (0.2–16.4)	0.3 (0.2–0.9)	<0.0001
CDS	5 (1–8)	5 (2–7)	0.96
FIB-4	2.9 (0.4–17.3)	1.7 (0.8–3.7)	<0.0001
FibroQ	2.8 (0.6–13.6)	3.9 (1.5–7.9)	0.06
Forns Score*	7.4 (2.3–10.0)	5.1 (3.1–8.5)	<0.0001
GUCI Index	2.1 (0.2–20.2)	0.4 (0.2–1)	<0.0001
King Score	38.3 (3.4–37.4)	7.7 (3.8–20.6)	<0.0001
Lok Index	0.4 (0–0.9)	0.4 (0.1–0.7)	0.33
PLF	na	2.5 (1.7–5.0)	na
ELF**	10.7 (7.9–14)	8.6 (6.8–10)	<0.0001

* Calculated in 33 patients for whom cholesterol values were available

** Calculated in 29 patients

Before treatment, none of the NITs showed any correlation with the most important histological features (i.e. fibrosis, activity, area of fibrosis). After treatment, some of the NITs (CDS, FIB-4, FibroQ, PLF) showed a correlation with the morphometric area of fibrosis, only; however, this correlation was lost at multivariate analysis **(Table 6).**

**Table 6:** Correlation between NITs and TE and histological features at univariate linear regression analysis.

	Pre-treatment	Post-treatment	Pre-Treatment	Post-Treatment	Pre-treatment		Post-Treatment	
Tests	p-value	p-value	p-value	p-value	r^2^	p-value	r^2^	p-value
APRI	0.69	0.38	-	0.56	0.00001	0.99	0.08	0.08
CDS	0.56	0.32	**-**	0.57	0.002	0.77	0.17	**0.04**
FIB-4	0.59	0.18	**-**	0.20	0.003	0.91	0.21	**0.003**
FibroQ	0.82	0.15	**-**	0.14	0.011	0.52	0.14	**0.01**
Forns Score	0.25	0.36	**-**	0.72	0.0003	0.90	0.10	0.06
GUCI Index	0.65	0.62	**-**	0.54	0.0007	0.95	0.08	0.07
King Score	0.58	0.40	**-**	0.32	0.14	0.14	0.14	**0.01**
Lok Index	0.42	0.06	**-**	0.23	0.03	0.25	0.08	0.07
PLF	0.17	0.53	**-**	0.09	0.0003	0.97	0.30	**0.0009**
ELF	0.17	0.83	**-**	0.69	0.11	0.06	0.08	0.11
TE	-	0.58	**-**	0.07	-	-	0.34	0.0004*

* Only TE maintained its statistical significance at multivariate analysis

TE: Transient Elastography

### Direct marker of fibrosis: ELF test

ELF test was assessed in 29 (76%) patients at baseline and after the SVR. Median post-treatment value was 8.6 (6.8–10.0) **(Table 3)**, without differences between regressors and non-regressors (8.6 *vs*. 8.4, p = 0.70) and across all stages of fibrosis (F1 *vs*. F2 *vs*. F3 *vs*. F4: p = 0.17) **(Fig 2)**. After the SVR, most patients had ELF test values below the 4 cut-offs suggested for the diagnosis of cirrhosis: 25 (86%) patients were < 9.3, 26 (90%) < 9.8, 29 (100%) < 10.3 and 29 (100%) <11.3; therefore ELF diagnostic accuracy could not be assessed according to the reference cut-offs. When trying to derive a new cut-off with the best sensitivity [60% (18.7–81.3)] and specificity [74% (43.4–87.4)], that resulted 8.1 in our cohort, it still carried a suboptimal performance (AUROC 0.63) for identifying residual cirrhosis **(Table 4)**. According to this cut-off, 5/18 (28%) patients were correctly classified as cirrhotics.

Before treatment, 25 (86%), 20 (69%) 17 (59%) and 5 (17%) patients were correctly classified as cirrhotics, according to the 9.3, 9.8, 10.3 and 11.3 cut-offs. Pre-treatment median ELF value was 10.7 (7.9–14), similar in regressors and non-regressors (10.6 *vs*. 10.8; p = 0.63). At the time of post-SVR LB, median values declined both in patients with and without cirrhosis regression (10.7 *vs*. 8.4, p<0.0001; 10.6 *vs*. 8.6, p = 0.0015) **(Table 5; Fig 4)**.

At linear regression analysis, ELF values did not correlate with any of the histological parameters as assessed at LB (activity, fibrosis, area of fibrosis), both at baseline and after the achievement of an SVR **(Table 6)**.

## Discussion

In this proof of concept study about diagnostic accuracy of serological assays for liver fibrosis, all tests failed to separate SVR patients with residual cirrhosis from minor levels of fibrosis. Cumulatively, the diagnostic accuracy of these tests by AUROC ranged from 0.51 and 0.75, whereas, individually, none had a cut-off value that could distinguish between different stages of fibrosis according to METAVIR.

In HCV patients, serological markers of liver fibrosis are becoming increasingly popular, as they have shown satisfactory accuracy for liver fibrosis staging, with AUROC ranging between 0.78 and 0.89 according to different studies **[29, 45]**. However, the performance of serological tests in HCV patients is lower than TE **[28,29,45]**. We previously demonstrated that TE failed to identify residual cirrhosis after the SVR using the classical *viremic* TE cut-off of 11.9 kPa **[24]**, speculating that the reduced TE accuracy in *non-viremic* patients could be the consequence of fibrosis remodeling occurring after the SVR. The present study focuses on this same subgroup of patients who were evaluated with both direct and indirect serological markers of fibrosis. Nine indirect tests failed to identify patients with residual cirrhosis, showing lower diagnostic accuracy in comparison with their performance reported in *viremic* patients **[29].** This poor diagnostic accuracy was in line with the reduced TE performance in this *non-viremic* population (AUROC serum assays *vs*. TE: 0.51–0.75 *vs*. 0.75) **[24]**. The loss in diagnostic accuracy was probably driven by the normalization of biochemical parameters that are considered in each test assessed, thus confirming a previous report by Sebastiani et al, who showed poor performance of APRI, AAR, Forns’ Index, Fibrotest and Fibroindex in 80 *viremic* patients with persistently normal transaminase values **[46]**. On the other hand, a recent study in 115 patients tested with APRI, FIB-4 and Forns Score 6 years after the SVR found increased values in parallel with histological fibrosis stage (p<0.0001), however with low specificity and PPV **[47]**. These findings discrepant from our study could be mainly explained by differences in patient demography, stage of fibrosis and cumulative analysis of residual F3 and F4 stages.

To overcome the pitfalls of a single-test approach in a relatively small study, we tested whether the combination of different serological tests and TE resulted in improved diagnostic accuracy. This notwithstanding, PLF, which combines four indirect markers of fibrosis and TE, showed poor diagnostic accuracy, although it carried the best performance among all tests. Differences observed in PLF values between regressors and non-regressors (p = 0.01) and across all stages of fibrosis (p = 0.01) were likely consequence of the inclusion of TE values in the test formula, since TE values significantly separated F4 from <F4 patients (p = 0.01) and correlated with post-SVR METAVIR stage (r = 0.56, p = 0.001). Not surprisingly, PLF diagnostic performance was close to that reported for TE in the same cohort in the previous study (AUROC PLF *vs*. TE: 0.75 *vs*. 0.77) **[24]**.

Since indirect serological tests are mostly based upon biochemical parameters of liver inflammation, which do not directly reflect extracellular matrix (ECM) remodeling, we also tested the diagnostic accuracy of ELF test, a direct marker of liver fibrosis that could be an ideal candidate for our study purposes. Indeed, ELF formula includes specific compounds of the ECM (TIMP-1, PIIINP, HA), which could be more sensitive to hepatic fibrosis rearrangement occurring after HCV eradication. Although we were not able to calculate ELF values for all the patients included in this study, we observed that these values were significantly reduced in post-SVR assessment compared to pre-treatment sampling. ELF diagnostic performance for identifying residual cirrhosis was sub-optimal in line with other indirect tests. Even the 8.1 derived cut-off did not further improve ELF diagnostic accuracy (AUROC 0.63). Another study in 81 SVR patients (22% with cirrhosis) found lower ELF values at follow-up than at baseline, while median ELF values in SVR patients were lower than in non-responder patients **[48]**. However, this study was flawed by the lack of a histological reference standard.

While a reduction of serum tests for fibrosis following an SVR has already been reported in HCV patients **[47,49]**, the present study has the merit to correlate serological tests with post-treatment stages of liver fibrosis. Indeed, identification of residual cirrhotics in SVR patients bears clinical interest, since cirrhotic patients who achieve an SVR following antiviral treatments are currently maintained under surveillance due to their residual risk of HCC **[17]**. A few years ago, Mallet and colleagues demonstrated that patients who achieved cirrhosis regression after an SVR remained free from liver-related complications in the following 5 years **[9]** whilst HCC developed in patients with persistent cirrhosis, only. Thus, assessment of post-SVR fibrosis stage might help individualizing the surveillance algorithm in patients with a pre-treatment diagnosis of cirrhosis who achieved viral eradication, although this individual approach is not recommended by international societies **[17]**. A point which needs clarifications is also whether serum assays of liver fibrosis predict long-term outcome and prognosis of SVR patients as they do with *non-viremic* patients **[41,50]**. By the same token, it could be of interest to identify post-SVR cut-offs of fibrosis tests predicting clinical events during surveillance. In our small study, we recorded no liver-related events over a 5-year follow-up, thus preventing the possibility to correlate post-SVR fibrosis stage with clinical outcomes, or to identify those patients at risk of complications according to their post-treatment values of serum tests. Due to the lack of robust studies assessing the accuracy of non-invasive tests in staging post-SVR fibrosis and/or predicting clinical events after HCV eradication, international guidelines do not recommend their use in tailoring the management of post-treatment follow-up **[45].**

We acknowledge weaknesses of our study that may ultimately affect the strength of our conclusions. No doubts that sample size is one such weakness, but it was to some extent the result of our stringent criteria of patient selection in terms of well-compensated patients fitting IFN-based therapy, who gave their informed consent to perform a second liver biopsy and the long term surveillance of 5 year on average post-SVR. In addition, for some of these patients Forns Score, PLF and ELF tests were not available, thus probably limiting the accuracy of their predictive value among this cohort. Finally, the retrospective design of the study prevented the dynamic study of serum tests of fibrosis. On the other hand, we think that these *cons* are counterbalanced by several strengths including the single center design in which liver biopsies were collected before treatment and 5 years following an SVR, and read by two expert pathologists blinded to any clinical features.

In conclusion, this study demonstrates that serological biomarkers of liver fibrosis failed to stratify SVR patients by degree of residual fibrosis and, most important, did not allow identification of patients with residual cirrhosis following an SVR.

## Abbreviations

HCV: Hepatitis C Virus
SVR: Sustained Virological response
IFN: Interferon
HCC: Hepatocellular Carcinoma
LB: Liver Biopsy
TE: Transient Elastography
NITs: Non Invasive Tests
APRI: AST to platelet ratio
CDS: Cirrhosis Discriminate Score
GUCI: Goteborg University Cirrhosis Index
PLF: Predicted Liver Fibrosis
ELF: Enhanced Liver Fibrosis
